# The implications of differential clustering for analysis of binary outcomes

**DOI:** 10.1186/1745-6215-14-S1-O41

**Published:** 2013-11-29

**Authors:** Chris Roberts, Steve Roberts, Eva Batistatou

**Affiliations:** 1 University of Manchester, Manchester, UK

## Introduction

Clustering may arise in clinical trials due to either randomisation in a cluster randomised trial, or due to treatment. Examples of treatment related clustering are activity classes or therapeutic support groups, where patients are nested in therapy group, or talking and physical therapies where patients are nested by therapist. In either setting the clustering effect may differ between treatments.

In this presentation we consider the implications of a differential clustering effect for analysis of binary outcome measures.

## Methods

For binary outcome measures possible methods of analysis include an adjusted test of proportions, summary measures analyses, or logistic models with random effects, GEE with an exchangeable correlation structure, or robust standard errors.

Issues related to consistency were investigated using numerical integration and by estimation of the treatment effect in large samples in which 800,000 subjects were simulated.

Small sample properties were investigated using simulation studies with 20,000 replications for each scenario and a range of studies sizes appropriate for estimation of small to medium treatment effect sizes.

## Results

Random coefficient models are found to lack consistency with other methods when estimating a null treatment effect. This has implications for test size. Small sample biases are also present for some methods. Asymmetry in the test statistic is found to have implications for test size and power.

## Conclusions

We do not recommend use of logistic random coefficient models for the analysis of clustered trials due to lack of consistency for a null effect.
